# Monitoring Animal Poisoning in Central Italy: Long-Term Trends and Spatial Distribution in the Abruzzo Region

**DOI:** 10.3390/ani16132064

**Published:** 2026-07-04

**Authors:** Antonio Petrini, Alice Menozzi, Carmine Merola, Giampiero Scortichini, Daria Di Sabatino, Luca Brugnola, Stefania Salucci, Elga Ersilia Tieri, Daniela Averaimo, Antonio Cocco, Giorgio Saluti, Giuseppe Gatti, Rachele Rocchi, Davide De Tiberis, Maria Laura Marchetti, Daniel Buldain, Pedro Zeinsteger, Pietro Badagliacca

**Affiliations:** 1 Istituto Zooprofilattico Sperimentale dell’Abruzzo e Molise “G. Caporale”, Campo Boario, 64100 Teramo, Italy; a.petrini@izs.it (A.P.); amenozzi@unite.it (A.M.); g.scortichini@izs.it (G.S.); d.disabatino@izs.it (D.D.S.); s.salucci@izs.it (S.S.); e.tieri@izs.it (E.E.T.); d.averaimo@izs.it (D.A.); a.cocco@izs.it (A.C.); g.saluti@izs.it (G.S.); g.gatti@izs.it (G.G.); r.rocchi@izs.it (R.R.); ddetiberis@unite.it (D.D.T.); piba@live.it (P.B.); 2 Department of Veterinary Medicine, University of Teramo, 64100 Teramo, Italy; 3 Gruppo Carabinieri Forestali Teramo, 64100 Teramo, Italy; luca.brugnola@carabinieri.it; 4 Facultad de Ciencias Veterinarias, Universidad Nacional de La Plata, La Plata 19100, Argentina; mlmarchetti@fcv.unlp.edu.ar (M.L.M.); danibuldain@gmail.com (D.B.); petzeins@gmail.com (P.Z.)

**Keywords:** animal poisoning, anticoagulant rodenticides, metaldehyde, pesticides, pets, wildlife

## Abstract

Both domestic and wild animals are frequently affected by accidental, intentional, or illegal poisoning globally, but data from some regions, including Italy, remain scarce. This study aimed to characterize animal poisoning events in a central Italian region over the period 2011–2020. A total of 2722 suspected cases were analyzed, of which 51.3% were confirmed positive for at least one poison. Among the 1397 confirmed cases, 863 animal carcasses and 534 poisoned baits were recorded, corresponding to 762 distinct poisoning events. The most frequently involved toxicants were metaldehyde, carbamates, and organophosphorus pesticides, followed by anticoagulant rodenticides and other compounds. Spatial analysis revealed distinct patterns in the use of toxicants. Sub-regional areas encompassing national and regional parks were primarily associated with agricultural pesticides, whereas urban areas were dominated by metaldehyde and anticoagulant rodenticides. These differences suggest that, in addition to their illegal nature, poisoning events may reflect underlying socio-environmental conflicts between humans and animals. The results highlight the value of an interdisciplinary approach to mitigate poisoning, involving coordinated efforts among scientific, veterinary, enforcement, and public stakeholders. Such strategies could reduce both the frequency and ecological impact of this illegal practice.

## Simple Summary

The poisoning of domestic and wild animals—whether accidental, deliberate, or unlawful—is a well-documented phenomenon in the global literature and continues to represent a significant risk for humans, animals, and the environment. In the present study, we investigated the temporal and spatial distribution of animal carcasses and suspected poisoned baits collected in the Abruzzo region, located in central Italy, and submitted to a public veterinary institute for necropsy, inspection, and toxicological analyses. The analysis of the temporal trend of cases and poisoning events between 2011 and 2020 highlights that animal poisoning remains an ongoing threat in this area. However, increasing public awareness and reporting may also have contributed to a higher rate of confirmed diagnoses. The spatial distribution analysis aimed to identify the types of toxic substances used in relation to different land-use contexts (e.g., urban areas versus mountainous areas within natural parks). This approach also helped to highlight the different types of human–animal conflicts underlying this illegal practice.

## 1. Introduction

Animal poisoning is a frequent occurrence that is currently receiving significant attention, driven by growing public sensitivity and awareness [1]. Depending on the underlying circumstances, these toxicological events can be categorized into three main drivers: accidental exposure, illegal practices, or deliberate criminal intent. While accidental cases often stem from environmental contamination or the misuse of domestic products, intentional and unlawful acts represent severe crimes that target both domestic species and wildlife [1]. Among these crimes, poisoning represents a deliberate act aimed at killing or attempting to kill an animal by releasing food preparations mixed with toxic substances or harmful materials (baits) into its habitat. There is a widespread consensus on the association between violent human behavior and acts of animal cruelty, including poisoning [2,3]. Furthermore, the release of poisoned baits in the environment, especially in urban areas, raises significant public health and safety concerns. A recent review by [4] provided a comprehensive overview of animal poisoning, distinguishing between intentional poisoning, accidental exposure to legally available substances, and secondary poisoning due to scavenging or predation on already poisoned animals.

Numerous chemical agents are commonly implicated in animal poisoning. These include carbamates, organophosphates (OPs), organochlorines (OCs), and pyrethroids, which are often available for agricultural use under legal restrictions or in smaller quantities for domestic gardening and veterinary applications [5,6]. Due to their large availability, some legally marketed products, such as molluscicides, rodenticides, and antifreeze, are frequently involved in both accidental and intentional poisoning incidents [7]. However, long-banned compounds such as strychnine and 2-methyl-4,6-dinitrophenol (DNOC) are also routinely investigated in suspected poisoning cases [8,9].

Acute poisoning typically refers to the onset of clinical signs within 24 h post-exposure [10]. The severity of symptoms depends on both the toxic dose and species-specific sensitivity. Clinical manifestations can range from non-specific signs such as vomiting, diarrhea, and hyperthermia, to severe neurological (e.g., ataxia, convulsions, depression, and hyperexcitability) and cardio-respiratory effects (e.g., dyspnea and shock), potentially resulting in sudden death. In many cases (particularly involving wildlife), animals are found dead with no prior clinical signs. Bleeding may be associated with anticoagulant rodenticide ingestion or as a secondary effect of pulmonary congestion during toxic shock, even with delayed onset [8,11,12].

Toxicological diagnosis typically involves analysis of biological matrices from symptomatic animals and tissue samples from carcasses. Necropsy findings of poisoned animals often include characteristic pathological features, such as intracavitary serous-hemorrhagic exudates associated with multiorgan congestive status and hyperemia of gastro-enteric mucosa. The presence of foreign materials and the detection of chemical odors in the gastrointestinal tract can provide useful guidance for subsequent toxicological analyses [13].

In recent years, several regional reports from both northern and southern Italy have contributed to a better understanding of poisoning patterns. These studies have documented either the proportion of confirmed cases among suspected submissions or the geographical distribution of specific toxic agents [14,15,16,17]. However, in central Italy, particularly in the Abruzzo region, there are still limited published data examining long-term trends in animal poisoning and the local distribution of cases involving both domestic animals and wildlife.

To address this gap, the present study analyzes laboratory-confirmed cases of animal poisoning recorded in the Abruzzo region between 2011 and 2020. The aim is to identify the toxic agents involved, describe the spatial distribution of poisoning events, and explore potential anthropogenic and environmental factors that may contribute to the persistence of this phenomenon.

## 2. Materials and Methods

### 2.1. Study Area, Sample Collection, and Case/ Event Definition

This study was carried out in the Abruzzo region, located in central Italy. More than one-third of the regional territory is designated as a protected Apennine area and includes several national and regional parks. Based on the morphological and ecological features of the territory—such as land use (agricultural versus forested areas), population density, the presence of hunting districts, truffle harvesting areas, and habitats of wild carnivores—the region was divided into eight sub-regional areas for analytical purposes. Specifically, the eight sub-regional areas were delineated in a GIS environment by aggregating whole municipal administrative boundaries based on their shared altimetric and ecological features.

Between 2011 and 2020, samples consisting of animal carcasses and suspected poisoned baits were submitted by local veterinarians to the Istituto Zooprofilattico Sperimentale dell’Abruzzo e del Molise (IZSAM), in accordance with ministerial guidelines. For each submission, information on the recovery site and individual animal data (including species and geographic coordinates) was recorded. The initial diagnostic assessment consisted of necropsy for carcasses and macroscopic examination for baits. Cases in which alternative causes of death—such as traumatic injuries or infectious diseases—were definitively established were considered negative. Carcasses and baits classified as suspected poisoning cases were subjected to further analytical investigations.

Toxicological analyses were performed on both domestic and wild animal carcasses, as well as on suspected poison baits. When gastric contents were unavailable, liver tissue was analyzed. In cases of suspected ethylene glycol poisoning, renal tissue was examined histologically to support the diagnosis. A confirmed toxicological diagnosis was established by correlating macroscopic and microscopic pathological findings with the analytical detection and quantification of toxic compounds within the gastric matrix or hepatic tissues.

A poisoning event was defined as one or more toxicologically confirmed cases occurring in the same or a nearby geographic location, involving the same toxic compound, and reported within a time interval not exceeding one week. For each event, the number of poisoned animals and/or contaminated baits was recorded. Affected species were classified as companion animals (dogs and cats), wild mammals, wild birds, livestock, or mixed species (i.e., events involving more than one taxonomic group).

Identified toxicants were grouped into seven categories: carbamates, organophosphates (OPs), metaldehyde, anticoagulant rodenticides (ARs), long-banned substances (including strychnine and 2-methyl-4,6-dinitrophenol [DNOC]), other toxicants (such as organochlorines (OCs) and infrequently detected harmful compounds), and mixed poisonings involving two or more of the above categories.

To assess the magnitude of poisoning events, a case-to-event ratio (case/event index) was calculated. A value of 1 corresponded to events involving a single case, whereas values greater than 1 indicated multiple poisoned animals and/or baits within the same event. This index was used to evaluate the severity and spatial distribution of poisoning episodes across the sub-regional areas and according to toxicant category.

### 2.2. Toxicological Investigations

Toxicological determinations were performed by means of liquid chromatography–tandem mass spectrometry (LC-MS/MS), gas chromatography–mass spectrometry (GC-MS), and gas chromatography with nitrogen phosphorus detector (GC-NPD) or electron capture detector (GC-ECD).

#### 2.2.1. Carbamates and Anticoagulant Rodenticides

The determination of carbamates (aldicarb, carbaryl, carbofuran, methiocarb, and methomyl) and anticoagulant rodenticides (brodifacoum, bromadiolone, coumachlor, coumatetralyl, coumarin, difenacoum, and warfarin) was carried out by LC–MS/MS. The sample (5 g from the liver and 1 g from the baits and stomach contents) was extracted with 20 mL of diethyl ether with the addition of nicarbazin and triphenyl phosphate as internal standards at the concentration of 10 mg/kg. After centrifugation, the organic phase was evaporated to dryness and the residue was dissolved in 1 mL of methanol. A 50 µL volume of re-dissolved extract was diluted with 450 µL of methanol and 500 µL of water containing 5 mm ammonium acetate and 1% acetic acid, and then, 10 µL of this solution was injected into an API 3000TM (Sciex, Framingham, MA, USA) triple quadrupole mass spectrometer, equipped with a turbo ion spray source, and the Series 200 HPLC systemTM (PerkinElmer Inc., Shelton, CT, USA) and software Analyst version 1.6.2 The ion source was operated in both positive and negative (only for bromadiolone) ionization modes using a spray voltage of ±5000 V and a temperature of 400 °C, while nitrogen was used as nebulizing gas (8 psi) and turbo gas (10 psi). The multiple reaction monitoring (MRM) approach was used for quantitative analysis. The separation was performed on a Gemini column C18 100 mm × 2 mm, 5 µm (Phenomenex, Torrance, CA, USA), using a mobile phase at 0.2 mL/min flow rate that consisted of methanol (A) and water (B), both containing 5 mm of ammonium acetate and 1% acetic acid in gradient elution mode: 0 min 100% B, 11 min 100% A, 23 min 100% A, 25 min 100% B, and 36 min 100% B. The monitored MRM transitions (*m*/*z* values) were as follows: aldicarb 208-89, carbaryl 202-127, carbofuran 222-123, methiocarb 243-169, methomyl 163-88, brodifacoum 523-291, bromadiolone 525-250, coumachlor 343-163, coumatetralyl 293-131, coumarin 147-91, difenacoum 446-179, and warfarin 309-163. For all carbamates and anticoagulants, the limit of quantification (LOQ) value was 0.020 mg/kg, while the mean recovery rates were in the range 60–130%, and repeatability standard deviation (RSDr) was between 8 and 20% at the 0.1 mg/kg level.

#### 2.2.2. OP and OC Pesticides

The OP [azinphos ethyl, azinphos methyl, chlorpyrifos, diazinon, dimethoate, fenthion, malathion, mevinphos, parathion ethyl, parathion methyl, phorate, and fenchlorphos (ronnel)] and OC pesticides (aldrin, dichlorodiphenyldichloroethane (DDD), dichlorodiphenyldichloroethylene (DDE), dichlorodiphenyltrichloroethane (DDT), dieldrin, endosulfan-alpha, endosulfan-beta hexachlorobenzene, hexachlorocyclohexane (α-HCH e β-HCH), heptachlor, heptachlor epoxide, lindane, and methoxychlor, mirex) were analyzed by GC-NPD and GC-ECD, respectively. The sample (1 g) was extracted with water (5 mL), acetone (10 mL), sodium chloride (1.5 g), and hexane (7.5 mL). After centrifugation, 5 mL of the upper layer was concentrated to 1 mL. A volume of 1 µL was injected into a GC-NPD TraceTM GC Ultra (Thermo Fisher Scientific, Waltham, MA, USA) with software Chrom-card version 2.4.1 (Thermo Fisher Scientific, Waltham, MA, USA). for OP pesticide analysis. The GC column was a DB-1 15 m × 0.25 mm × 0.20 mm (J & W, Agilent, Santa Clara, CA, USA) using an oven temperature program of 98 °C for 2 min that was then increased to 145 °C at 20 °C/min and maintained for 5 min, and finally increased to 280 °C at 4.5 °C/min and held for 7 min. The injector temperature was 265 °C, detector temperature 285 °C, and helium flow rate 1 mL/min. A volume of 1 µL was injected into a GC-ECD CP-3600TM (Varian Analytical Instruments, Walnut Creek, CA, USA) with software Chrom-card version 2.3.3 (Thermo Fisher Scientific, Waltham, MA, USA). for OC pesticides analysis. The GC column was a DB-1701 15 m × 0.25 mm × 0.20 mm (J & W, Agilent, Santa Clara, CA, USA); the oven temperature program was 98 °C for 2 min, an increase to 160 °C at 20 °C/min, maintenance for 1 min, then an increase to 240 °C at 1.8 °C/min, holding for 0.1 min, finally an increase to 270 °C at 20 °C/min, and holding for 7 min. The injector temperature was 270 °C, detector temperature 320 °C, and helium flow rate 1 mL/min.

For organochlorine pesticides, the LOQ was 0.010 mg/kg, the mean recoveries were in the range 95–114%, and RSDr varied from 6% to 22% at 0.1 mg/kg. For organophosphate pesticides, the LOQ was 0.002 mg/kg, the mean recoveries fluctuated in the range 60–115%, and RSDr varied from 11% to 20% at 0.1 mg/kg.

#### 2.2.3. Pyrethroids, Oxamyl, and DNOC

Pyrethroids (cyfluthrin, cypermethrin, deltamethrin, and permethrin), oxamyl, a carbamate, and 4,6-dinitro-ortho-cresol (DNOC) were analyzed using a GC-MS method. Briefly, 0.5 g of sample was extracted by a mixture of water (5 mL), acetone (10 mL), sodium chloride (1.5 g), and hexane (7.5 mL). After centrifugation, 5 mL of the upper layer was concentrated to 1 mL, and 1 µL was injected in a GC-MS system constituted of a TraceTM GC 2000 gas chromatograph coupled to a DSQ single quadrupole mass spectrometer (Thermo Fisher Scientific spa, Rodano, MI, Italy) with software XCalibur version 2.0.7 (Thermo Fisher Scientific, Waltham, MA, USA). Chromatographic separation was performed on a DB-5 MS 30 m × 0.25 mm × 0.25 mm capillary J & W GC columnTM (Agilent Technologies, Santa Clara, CA, USA). The oven initial temperature was programmed at 98 °C and increased to 160 °C at 25 °C/min, then to 210 °C at 4 °C/min, and finally to 280 °C at 10 °C/min with a maintenance of 15 min. The injector temperature was 250 °C, ion source temperature 260 °C, flow rate 1 mL/min for the carrier gas helium. The analytes were determined by single ion monitoring (SIM) on three ions with the following *m*/*z* values: cyfluthrin 206, 165, and 163; cypermethrin 234, 206, and 149; deltamethrin 176, 107, and 89; permethrin 183, 165, and 163; oxamyl 145, 98, and 72; and DNOC 198, 121, and 105.

For pyrethroids, oxamyl, and DNOC, recovery and RSDr were not estimated since a semi-quantitative approach was adopted. Their LOQ values were between 0.1 mg/kg and 0.5 mg/kg.

#### 2.2.4. Strychnine

Strychnine was analyzed by GC-MS after extraction of the sample (2 g for liver and 1 g for other matrices) with 20 mL of dichloromethane and 20 mL of sodium hydroxide 1 M water solution. The organic phase was separated by centrifugation, and 1 µL was injected in GC-MS. The GC column was a DB-5 MS 30 m × 0.25 mm × 0.20 mm (J & W, Agilent, Santa Clara, CA, USA) using an oven temperature program of 90 °C for 1 min, then an increase to 320 °C at 40 °C/min, and maintenance for 8 min. The injector temperature was 270 °C, ion source temperature 250 °C, and flow rate 1 mL/min for the carrier gas helium. The analyte was determined by SIM (ions with *m*/*z* 334 and 162). For strychnine, the LOQ values were 0.1–0.4 mg/kg depending on the matrix. The mean recovery was between 99% and 115%, while RSDr was found to be in the range 6–22% at 0.1 mg/kg.

#### 2.2.5. Metaldehyde

Metaldehyde was determined by GC-MS after extracting 2 g of sample with 40 mL of chloroform. A volume of 1 µL was injected in GC-MS using a DB-5 MS 30 m × 0.25 mm × 0.20 mm (J & W, Agilent, Santa Clara, CA, USA) column. The GC oven temperature program was 85 °C for 3 min, then an increase to 310 °C at 40 °C/min, and maintenance for 5 min. The injector temperature was 270 °C, ion source temperature 250 °C, and helium flow rate 1 mL/min. The analyte was determined by SIM (ions with *m*/*z* 131 and 89).

For metaldehyde, the LOQ was 5.0 mg/kg, the mean recovery was between 87% and 97%, and RSDr fell in the range 8–11% at the 10 mg/kg level.

#### 2.2.6. Zinc Phosphide

Zinc phosphide was analyzed by GC coupled to a nitrogen phosphorus detector (NPD). After treatment of the sample (2 g) with 5.5 mL of orthophosphoric acid 10% water solution in a vial for headspace gas analysis, phosphine resulting from zinc phosphide was injected into a GC-NPD system Clarus 680TM (PerkinElmer, Shelton CT, USA) with software Totalchrom version 6.3.2 (PerkinElmer, Shelton CT, USA). The GC column was a GS-Q 30 m × 0.53 mm megabore (J & W, Agilent, Santa Clara, CA, USA). The oven temperature program was 45 °C for 10 min, then an increase to 230 °C at 30 °C/min, and maintenance for 10 min. The injector temperature was 230 °C, detector temperature 280 °C, and helium flow rate 1 mL/min. For zinc phosphide, the LOQ was 2.5 mg/kg, and recovery and RSDr were not estimated since a semi-quantitative approach was adopted.

### 2.3. Statistical Analysis

Territorial data—including municipalities, population density, surface area, elevation, and morphology—were obtained from official census records provided by the Italian National Institute of Statistics (ISTAT; www.istat.it). Individual cases were georeferenced using the World Geodetic System 1984 (WGS84) and mapped with the Universal Transverse Mercator projection (UTM Zone 33). Clusters of cases were assigned the coordinates of the first recorded case.

Descriptive statistics, including central tendencies, ratios, indices, and 95% confidence intervals, were calculated using Microsoft Excel™ (version 2021). To assess whether the number of animals involved per poisoning event differed among poisoning categories and geographical areas, we compared the observed number of cases in each category with the number expected under the assumption of a common case-to-event ratio across categories. The number of poisoning events was used as the exposure variable. An overall chi-square goodness-of-fit test was performed to evaluate heterogeneity among categories. When significant, post hoc pairwise comparisons of case-to-event rates were conducted using exact conditional tests for two Poisson rates, and *p*-values were adjusted for multiple comparisons using the Holm correction. The Mann–Kendall trend test was applied to the annual number of confirmed poisoning cases recorded between 2011 and 2020 to describe the temporal pattern of positivity.

## 3. Results

Table 1 presents the temporal distribution of the total number of suspected poisoning cases. Between 2011 and 2020, a total of 2722 cases were analyzed, of which 51.32% tested positive for at least one poison. The annual number of confirmed poisoning cases showed considerable interannual variability, ranging from 75 cases in 2012 to 215 cases in 2018. A Mann–Kendall trend test nevertheless indicated a significant positive temporal trend over the study period (Kendall’s τ = 0.60, *p* = 0.017), supporting an overall increase in the number of confirmed cases between 2011 and 2020, although the pattern was not strictly monotonic.

Table 2 summarizes the confirmed poisoning events by toxicological category. Among the 1397 positive cases, 863 animal carcasses and 534 baits were identified, corresponding to 762 distinct poisoning events. The highest number of events was associated with metaldehyde, carbamates, and organophosphorus pesticides, followed by anticoagulant rodenticides and other toxic agents. Significant differences (adjusted *p* < 0.05) indicated higher case/event ratios for strychnine and DNOC, mixed poisonings, and carbamates compared with several other categories. In particular, strychnine and DNOC, and mixed poisonings consistently showed higher ratios than metaldehyde, OP pesticides, anticoagulant rodenticides, and other poisonings, while carbamates showed elevated ratios compared with most categories except strychnine and DNOC. Dogs and cats were involved in 486 events, including 429 dogs and 163 cats, either as single species affected or concurrently within the same event. An additional 116 companion animals (dogs or cats) were involved in 79 events in which poisoned baits were also recovered. Wild carnivores were implicated in 38 events. Overall, 534 poisoned baits were confirmed. Of these, 283 were recovered in 132 events not associated with poisoned animals, whereas 251 were linked to 96 events involving different animal species categories.

**Figure 1:**
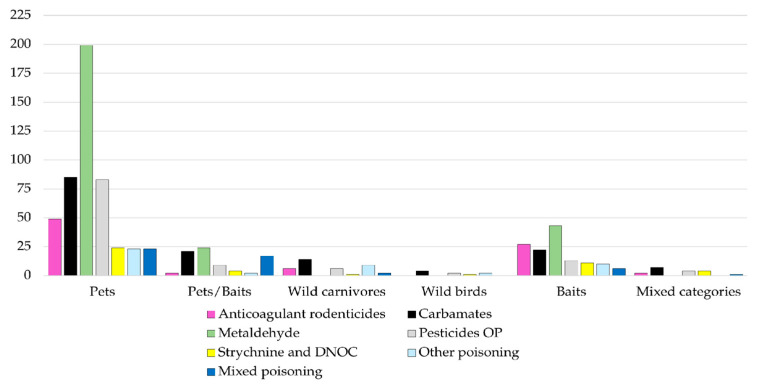
Distribution of poisoning events according to animal categories, baits, and toxicological groups.

Figure 1 illustrates the distribution of poisoning events according to animal categories, baits, and toxicological groups. Companion animals were predominantly involved in poisoning events caused by metaldehyde, followed by carbamates and organophosphorus pesticides. In contrast, wild carnivores were most frequently associated with carbamate-related events. Specifically, dogs and cats were the primary species involved in the observed events, both individually (dogs: n = 421; cats: n = 157) and in combination with each other (n = 14) or alongside recovered baits (n = 116). Wildlife was implicated in 117 poisoning cases, mostly involving foxes (n = 65) and wolves (n = 23), and to a lesser extent, mustelids (n = 6). Four cases were recorded in the Marsican brown bear (*Ursus arctos marsicanus*) and among wild birds (18 poisoned animals); nine cases involved Griffon vultures; and the remainder affected red kites (*Milvus milvus*), buzzards (*Buteo buteo*), and corvids. Regarding baits, the majority of poisoning events were also linked to metaldehyde exposure. Among the identified materials used as bait or found in stomach contents, the most frequent matrices were, in descending order, miscellaneous foods of animal origin, minced meat or meatballs, fresh sausages, frankfurters/cured meats, commercial pet food, and kitchen scraps. Although less common, other delivery methods were also observed, including the use of animal carcasses as bait, complex food preparations adulterated with hazardous objects, or baits prepared with a calibrated dosage of toxic substances. Poisoning incidents were reported in 184 of the 305 regional municipalities (60.3%), corresponding to 0.13 cases/km^2^/year.

**Figure 2:**
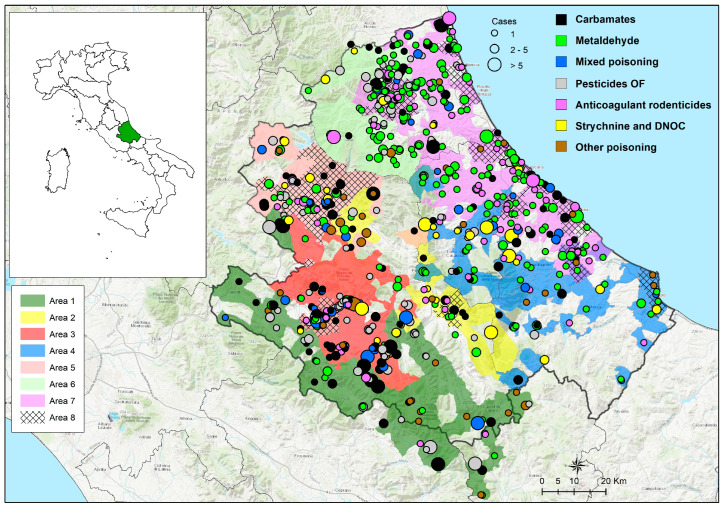
Spatial distribution of poisoning events in the Abruzzo region areas (2011–2020). Circle size reflects the number of cases per event.

Table 3 and Figure 2 summarize the distribution of events and associated cases by type of poisoning across the eight sub-regional areas.

Sub-regional areas differed significantly in the number of animals involved per poisoning event, as shown by the overall chi-square goodness-of-fit test comparing observed and expected cases under the assumption of a constant case/event ratio across areas (chi-square = 74.47, df = 7, *p* = 1.84 × 10^−13^). Based on the aggregated area level data, the overall case/event index was 1.85. Area 1—corresponding to the Parco Nazionale d’Abruzzo, Lazio e Molise (PNALM)–Liri Valley—showed the highest index, with 252 cases in 86 events, corresponding to 2.93 cases/event, and was significantly higher than expected after Holm correction (*p* < 0.001).

Area 2 (National Parks interface; index = 2.00), Area 3 (Sirente–Velino–Fucino; index = 1.99), and Area 4 (Majella Park–Adriatic Valleys; index = 1.85), together with Area 1, accounted for 49.7% of all recorded cases. Pairwise post hoc comparisons of Poisson rates confirmed that Area 1 had a significantly higher case/event rate than Areas 3, 4, 5, 6, 7, and 8. In Area 1, high-intensity clusters were primarily associated with carbamates, organophosphates (OPs), and mixed poisonings, with case/event indices of 3.88, 2.58, and 7.25, respectively. Area 3 (Sirente–Velino–Fucino) showed elevated indices for carbamates (2.46), strychnine or DNOC (3.29), and mixed poisonings (2.42). In Areas 2 and 4, poisonings were mainly linked to strychnine/DNOC (indices = 4.00 and 3.67, respectively) and mixed toxic agents (indices = 2.60 and 7.50, respectively).

In contrast, the urban areas, Area 7 and Area 8, which accounted for 37.0% of total cases, showed significantly lower case/event indices than expected, with values of 1.51 and 1.61, respectively. When Areas 1–6 were grouped and compared with the urban areas 7–8, the case/event rate was significantly higher in Areas 1–6 than in Areas 7–8, 2.07 versus 1.56 cases/event, respectively (exact conditional test for two Poisson rates, *p* = 3.57 × 10^−7^). In these areas, the metaldehyde-specific index was 1.43 and 1.26, respectively. Furthermore, anticoagulant rodenticides (ARs) were also predominantly recorded in urban centers (Area 7 and 8 indices = 2.25 and 1.00, respectively).

## 4. Discussion

This study analyzed animal poisoning data collected in the Abruzzo region between 2011 and 2020. The phenomenon proved to be widespread, with poisoning events reported in 60.3% of the region’s municipalities during the study period. Such spatial distribution suggests that the issue may reflect a broader pattern potentially affecting other regions of central Italy.

Between 2011 and 2020, a total of 2722 suspected poisoning cases were recorded, with an overall confirmation rate of 51.3%. The temporal trend indicated that confirmed cases tended to increase over the study period, despite marked year-to-year fluctuations. This upward trend indicates growing public awareness of the phenomenon and suggests that awareness-raising initiatives and prevention measures may have improved case detection and reporting. In practice, this higher notification rate and the more effective resolution of cases were driven by key institutional advancements: specifically, the deployment of specialized forestry units utilizing trained anti-poison canine teams to locate baits and carcasses, and the rigorous standardization of diagnostic procedures for both necropsy and chemical analyses.

Overall, 762 poisoning events were identified, involving 863 animals and 534 poisoned baits. Dogs and cats accounted for 74.1% of affected animals. Events in which poisoned baits were recovered without associated animal casualties represented 17.3% of cases, while incidents involving exclusively wildlife accounted for 6.2%. The predominance of companion animals among poisoning victims is consistent with previous reports from Italy [14,18,19] and other European countries [20]. Among the protected species, the red fox was the most frequently confirmed case of poisoning, agreeing with data from northern Italy and other European countries [18,19,21].

Excluding cases involving multiple active substances, 78.4% of the toxicants identified in confirmed poisoning events were represented by the following molecules: metaldehyde, aldicarb, phorate, bromadiolone and brodifacoum, strychnine, DNOC, and endosulfan. These compounds accounted for the majority of single-substance poisonings detected during the study period. Comparable toxicological patterns have been described in other Italian regions. In northeastern Italy, approximately 75% of poisoning events were attributable to the following group of compounds: metaldehyde, anticoagulant rodenticides (ARs), aldicarb, carbofuran, endosulfan, and strychnine [18]. In the Piemonte region, the predominant substances were ARs, metaldehyde, endosulfan, and methomyl [22], whereas in Lombardia and Emilia-Romagna, the most frequently detected toxicants included ARs, endosulfan, methomyl, methamidophos, strychnine, and carbofuran [19].

With the exception of oxamyl and metaldehyde, all active substances identified in the present investigation are currently listed as non-approved in the EU Pesticides Database under Regulation (EC) No 1107/2009. Notably, the re-emergence of DNOC as a causative agent of animal poisoning has also been recently documented in Italy [8].

The methodological approach adopted aimed to quantify poisoning events by distinguishing isolated incidents from serial occurrences (i.e., clusters). Events were analyzed within sub-regional areas defined according to orographic characteristics, land use, and population density. Conceptually, each event, whether isolated or repeated, was interpreted as a potentially intentional act, possibly constituting a criminal offence attributable to one or more perpetrators. Regardless of whether repeated events were committed by the same individual or by different individuals using similar toxicants, this study sought to explore the underlying socio-territorial conflicts potentially driving the phenomenon. This was achieved by assessing associations between poisoning events, toxicant categories, event intensity within specific areas, and local environmental and demographic features. The case-to-event ratio was used as an indicator of poisoning intensity for a given toxicant within a defined area.

The overall regional index was 1.83 cases per event. Sub-regional areas exceeding this threshold included Area 1 (PNALM–Liri Valley; index = 2.78; *p* < 0.001), Area 2 (National Park interface; index = 2.0), Area 3 (Sirente–Velino–Fucino; index = 2.0), and Area 4 (Majella Park–Adriatic Valleys; index = 1.85). These areas accounted for 61.3% of carbamate-related cases, 59.1% of organophosphate poisonings, 82.7% of strychnine/DNOC cases, 62.3% of mixed-toxicant events, and 68.3% of poisonings caused by other substances. Conversely, in the urban center sub-regions (Areas 7 and 8), where metaldehyde and anticoagulant rodenticides (ARs) predominated (56.8% and 66.3% of events, respectively), the case-to-event ratios were significantly lower (1.4 and 1.8, respectively; *p* < 0.05).

While accidental exposure can occur in urban settings due to the negligent or unintentional misuse of authorized products, the situation differs in other contexts. There, deliberate poisoning persists as an illegal yet widespread practice globally, driven chiefly by the intent to eradicate targeted animal species. Because poison is most commonly administered through the placement of toxic baits (e.g., pieces of meat, carcasses, or eggs) in open environments, its effects are often indiscriminate. Consequently, the illegal use of poisoned baits may impact a wide range of non-target species, including domestic animals; wildlife; and, in some cases, humans. Such practices are frequently associated with underlying conflicts between people and animals or among different human stakeholders [23]. Multiple factors contribute to the use of poisoning within the context of human–animal conflicts. Cultural traditions, historical attitudes, and socioeconomic conditions have all been linked to the persecution of wildlife. In particular, long-standing practices in rural communities may perpetuate the perception of certain species as threats to livelihoods or safety. Conversely, broader economic transitions, such as the shift from agriculture-based economies to industrial and technology-oriented sectors, may reduce reliance on traditional rural practices and potentially contribute to a decline in the illegal use of poison [23].

At least three main types of conflict were hypothesized as the underlying drivers of animal poisoning in the Abruzzo region. These constitute inferential assessments based primarily on spatial associations and contextual evidence, rather than direct investigations into the perpetrators.

The first relates to general aversion toward animals, stemming from fear or hostility and linked to perceived nuisances or disturbances. This dynamic applies to both domestic species and wildlife, particularly due to their close proximity to urban centers. Such tensions may escalate into intentional poisoning, particularly in densely populated or agricultural–hilly landscapes, including urban centers. In these areas, poisoning profiles were primarily characterized by the use of readily available compounds, such as metaldehyde and ARs. However, the improper use of legally available products for gardening or rodent control suggests that some single-case events involving these substances may fall within a grey area between accidental and intentional poisoning. Despite the potential intentional use of ARs in bait preparation (primary exposure), non-target animals can be exposed indirectly (secondary exposure) by feeding on poisoned target species, such as rodents and birds [24]. Furthermore, tertiary exposure occurs when an apex predator consumes a non-target animal affected by secondary exposure [24]. As previously documented in red foxes, the widespread application of ARs in urban and suburban areas has heightened concerns regarding both human and domestic/wild animal exposure [25]. Specifically, bromadiolone and brodifacoum were the most frequently detected AR compounds in the livers of Italian red foxes, also exhibiting the highest residue concentrations. This link is further supported by the Corine Land Cover map, which revealed that 10 out of the 13 positive samples originated from artificial areas, thereby highlighting the compounding effect of urbanization on AR exposure risk [25].

The second type of conflict concerns tensions arising from the presence of wild canids and broader human–wildlife interactions, particularly in areas where livestock farming interfaces with protected natural areas. This dynamic was mainly associated with Areas 1 and 3 (PNALM–Liri Valley and Sirente–Velino–Fucino). In these territories, the transition between intensively cultivated areas (e.g., the Fucino Plain) and protected mountainous landscapes is particularly narrow, creating a sensitive agro-forestry interface. Here, poisonings were predominantly linked to carbamates and organophosphates, which accounted for approximately 50% of events and displayed high case-to-event ratios, especially in municipalities located above 600 m a.s.l. Given their agricultural use and high toxicity, the involvement of these compounds strongly suggests deliberate and repeated criminal actions. Notably, more than 70% of confirmed wildlife poisoning cases originated from these areas.

The presence of protected areas has been identified as an additional factor potentially influencing the illegal use of poison. Although protected status might be expected to deter poisoning activities, through enhanced surveillance, stricter enforcement, and targeted awareness campaigns, legal conservation measures can, in some contexts, generate resentment among local communities. Such hostility may arise when conservation policies are perceived as restricting traditional land uses or limiting economic opportunities, thereby exacerbating conflicts between stakeholders and wildlife [23].

The third hypothesized conflict relates to competition over economically valuable natural resources, particularly truffle harvesting, a dynamic that may also extend to hunting grounds. With the exception of urban centers, all sub-regional areas include truffle harvesting sites regulated under regional legislation. The deliberate and repeated placement of poisoned baits in these contexts appears aimed at eliminating competitors’ truffle dogs to secure exclusive access to productive grounds. Although potentially present in multiple areas, this pattern was mainly observed in serial poisoning cases involving dogs and baits in the Adriatic valleys of Majella Park (Area 4) and in the internal interfaces of the three national parks. These events were characterized by high case-to-event ratios and the use of long-banned substances, particularly strychnine and DNOC. The high toxicological potency of these compounds likely explains the elevated number of cases associated with single poisoning events.

Although this study analyzed comprehensive temporal data on animal poisoning in the Abruzzo region, the research carries several limitations. These include its retrospective nature, the lack of GIS software utilization to map spatial distribution, and the inclusion of the year 2020, during which the COVID-19 pandemic and its associated restrictions may have led to an underestimation of the total number of cases. Moreover, the lack of a universal toxicological threshold for all cases might introduce a degree of misclassification.

## 5. Conclusions

The findings of this study confirm that animal poisoning remains a widespread and persistent issue in the Abruzzo region, constituting a serious form of criminal activity affecting both wildlife and companion animals, with significant ecological, public health, and legal consequences. Despite ongoing mitigation efforts, no clear decline in the incidence of poisoning was observed over the study period (2011–2020). However, the increased detection of poisoned baits in recent years indicates that surveillance and preventive measures, particularly the use of detection dog units, are producing measurable outcomes in reducing animal mortality. Nonetheless, the repeated occurrence of poisoning events in specific sub-regional hotspots highlights the persistence of unresolved socio-territorial conflicts.

A long-term, effective response to animal poisoning requires enhanced institutional coordination among regional authorities, judicial institutions, veterinary laboratories, environmental enforcement agencies, and protected area administrations. The findings of this study offer crucial practical implications for future interventions. Specifically, for surveillance programs, the identified sub-regional hotspots provide a data-driven framework to optimize resource allocation, allowing for targeted deployments of anti-poison dog units where the risk is highest. For wildlife conservation strategies, the results potentially underscore that long-term species preservation depends heavily on mitigating underlying socio-territorial conflicts at the borders of protected areas. Finally, regarding veterinary public health, the standardization of toxicological procedures and structured data collection are essential to establish reliable environmental health risk assessments. Key components of a comprehensive strategy include shared protocols for crime scene management; standardized toxicological procedures; raising public awareness; clear legal frameworks; and consistent enforcement measures, including prohibitions and confiscations, aimed at disrupting this illegal practice.

## Figures and Tables

**Table 1:** Temporal distribution of suspected animal poisoning in the Abruzzo region between 2011 and 2020.

Year	Positive Cases (Carcasses/Baits)		Negative Cases (Carcasses/Baits)	
	*n*	%	*n*	%
2011	86 (66/20)	62.77 (48.18/14.60)	51 (49/2)	37.23 (35.77/1.46)
2012	75 (61/14)	43.60 (35.47/8.14)	97 (88/9)	56.40 (51.16/5.23)
2013	106 (70/36)	51.46 (33.98/17.48)	100 (91/9)	48.54 (44.17/4.37)
2014	160 (104/56)	73.39 (47.71/25.69)	58 (45/13)	26.61 (20.64/5.96)
2015	161 (125/36)	47.92 (37.20/10.71)	175 (129/46)	52.08 (38.39/13.69)
2016	126 (76/50)	67.02 (40.43/26.60)	62 (47/15)	32.98 (25/7.98)
2017	168 (105/63)	48.84 (30.52/18.31)	176 (140/36)	51.16 (40.7/10.47)
2018	215 (95/120)	55.99 (24.74/31.25)	169 (130/39)	44.01 (33.85/10.16)
2019	109 (81/28)	27.66 (20.56/7.11)	285 (266/19)	72.34 (67.51/4.82)
2020	191 (80/111)	55.69 (23.32/32.36)	152 (141/11)	44.31 (41.11/3.21)
2011–2020	1397 (863/534)	51.32 (31.71/19.62)	1325 (1126/199)	48.68 (41.37/7.31)

**Table 2:** Number of poisoning events by toxicological category and corresponding positive cases in the Abruzzo region.

Type of Poisoning *	Events of Poisoning			Poisoned Animals			Poisoned Baits			Index Case/Event
	N	%	Average (CI 95)	n	%	Average (CI 95)	n	%	Average (CI 95)	
Carbamates	153	20.1	15.3 (12.7–17.9)	212	24.6	21.2 (15.4–27.0)	147	27.5	14.7 (5.8–23.6)	2.35
Metaldehyde	266	34.9	26.6 (20.3–32.9)	259	30	25.9 (20.2–31.6)	95	17.8	9.5 (5.1–13.9)	1.33
OP Pesticides	117	15.4	11.7 (10.1–13.3)	152	17.6	15.2 (11.7–18.7)	46	8.61	4.6 (1.2–8.0)	1.69
Anticoagulant rodenticides	86	11.3	8.6 (6.0–13.1)	64	7.42	6.4 (5.1–10.9)	82	15.4	8.2 (0.0–18.6)	1.70
Strychnine and DNOC	45	5.91	4.5 (3.5–6.5)	66	7.65	6.6 (4.6–10.1)	73	13.7	7.3 (4.0–14.3)	3.09
Other poisonings	46	6.04	4.6 (2.7–6.5)	38	4.4	3.8 (2.0–5.6)	25	4.68	2.5 (0.3–4.7)	1.37
Mixed poisonings	49	6.43	4.9 (3.4–6.4)	72	8.34	7.2 (4.8–9.6)	66	12.4	6.6 (4.1–12.4)	2.82
Total	762	100	76.2 (64.6–87.8)	863	100	86.3 (73.7–98.9)	534	100	53.4 (31.0–75.8)	1.83

* Among the detected carbamates, aldicarb was the predominant substance (106/153). Regarding organophosphate (OP) pesticides, phorate was identified most frequently (100/117), while brodifacoum and bromadiolone were the most common substances found among anticoagulant rodenticides (ARs) (68/86). Additionally, endosulfan emerged as the primary agent within the ‘other poisonings’ category (13/46). Other poisonings include organochlorines (OCs), arsenic, diethyltoluamide (DEET), zinc phosphide, paracetamol (acetaminophen), copper, and ethylene glycol. Mixed poisoning refers to the simultaneous presence of two or more different categories of toxicants within the same carcass or bait involved in a single poisoning event.

**Table 3:** Distribution of events and corresponding cases (values in parentheses) according to toxicological category across the eight sub-regional areas.

	Area 1	Area 2	Area 3	Area 4	Area 5	Area 6	Area 7	Area 8
Carbamates	32 (124)	8 (12)	24 (64)	12 (20)	14 (26)	12 (16)	18 (38)	31 (59)
Metaldehyde	13 (18)	9 (11)	12 (17)	37 (44)	11 (15)	33 (45)	81 (116)	70 (88)
OP Pesticides	24 (62)	5 (7)	29 (40)	6 (8)	3 (6)	18 (31)	15 (24)	17 (20)
ARs *	3 (5)	2 (2)	6 (6)	10 (10)	3 (4)	5 (17)	21 (21)	36 (81)
Strychnine/DNOC	5 (9)	3 (11)	7 (23)	18 (72)	5 (12)	2 (3)	3 (6)	2 (3)
Other poisonings	5 (5)	5 (10)	5 (14)	5 (5)	3 (3)	2 (4)	5 (5)	8 (8)
Mixed poisoning	4 (29)	2 (15)	12 (29)	5 (13)	2 (4)	0 (0)	12 (24)	12 (24)
Total	86 (252)	34 (68)	95 (193)	93 (172)	41 (70)	72 (116)	155 (234)	176 (283)
Index case/event	2.78	2	1.99	1.85	1.71	1.61	1.51	1.61

* ARs: anticoagulant rodenticides. Area 1: National Park of Abruzzo, Lazio, and Molise (PNALM)–Liri Valley, including the surrounding buffer zones and the southwestern regional border. Area 2: National Parks Interface, including internal corridors between major protected areas. Area 3: Sirente–Velino–Fucino, comprising the Sirente–Velino Regional Park and the agricultural plain of Fucino. Area 4: Majella National Park and Adriatic Valleys. Area 5: Western sector of the National Park of Gran Sasso and Monti della Laga (PNGSML). Area 6: Eastern sector of the PNGSML. Area 7: Inland areas of the Teramo, Pescara, and Chieti provinces. Area 8: Urban centers of Abruzzo provinces, including their immediate periurban suburbs and densely populated coastal municipalities.

## Data Availability

Data will be made available on request.
